# Actual and Perceived Units of Alcohol in a Self-Defined “Usual Glass” of Alcoholic Drinks in England

**DOI:** 10.1111/acer.12046

**Published:** 2012-12-20

**Authors:** Sadie Boniface, James Kneale, Nicola Shelton

**Affiliations:** UCL Research Department of Epidemiology and Public HealthLondon, United Kingdom; UCL Department of GeographyLondon, United Kingdom

**Keywords:** Alcohol Drinking, Public Health, Health Surveys

## Abstract

**Background:**

Several studies have found participants pour more than 1 standard drink or unit as their usual glass. This is the first study to measure actual and perceived amounts of alcohol in a self-defined usual glass of wines and spirits in the general population.

**Methods:**

Participants were a convenience sample of adults who drink alcohol or who pour drinks for other people (*n* = 283, 54% women) at 6 sites in South East England. The survey was face to face and comprised a self-completion questionnaire and pouring task. Estimation accuracy, categorised as correct (±0.5 units), underestimate (>0.5 units), or overestimate (>0.5 units) was the main outcome.

**Results:**

The mean number of units poured was 1.90 (SD 0.80; *n* = 264) for wine and 1.93 (SD 0.78; *n* = 201) for spirits. The amount of alcohol in a self-defined usual glass was estimated in 440 glasses (248 wine and 192 spirits). Overestimation took place in 42% glasses of spirit poured and 29% glasses of wine poured, and underestimation in 17 and 19%, respectively. Multinomial logistic regression found volume poured to be significantly associated with underestimating both wines and spirits, and additionally for wine only, belonging to a non-white ethnic group and being unemployed or retired. Not having a university degree was significantly associated with overestimating both drink types.

**Conclusions:**

This study is the first in the general population and did not identify systematic underestimation of the amount of alcohol in a self-defined usual glass. Underestimation is significantly associated with volume poured for both drink types; therefore, advocating pouring smaller glasses could reduce underestimation of alcohol consumption.

A systematic review (protocol available from lead author) of 12 previous studies found that participants often pour more than 1 standard drink or unit as their usual glass of alcoholic beverages (Banwell, 1999; Carruthers and Binns, 1992; Gill and Donaghy, 2004; Gill et al., 2007; Gual et al., 1999; Kaskutas and Graves, 2001; Kerr et al., 2005, 2008; Lemmens, 1994; Nayak et al., 2008; Wilkinson et al., 2011; Wilson, 1981). A single study reports investigating the perceived number of standard drinks poured in addition to measuring participants' self-defined usual glass. This study identified that participants underestimated on average by 23% in men and 16% in women and was conducted in Australia among 65- to 74-year olds only (Wilkinson et al., 2011). Two further US studies have found that college students pour significantly more than 1 standard drink when they are asked to pour 1 standard drink (White, 2005; White et al., 2003).

This inaccuracy arises due to underestimation of beverage volumes or strengths (as measured by alcohol by volume [ABV]). This particularly concerns drinks purchased from the off-trade (i.e., consumption taking place away from licensed premises) where beverages are not necessarily served in fixed measures as they are in the on-trade (bars, restaurants, etc.) in the United Kingdom. Two-thirds of all U.K. alcohol sales in 2010 were in the off-trade (British Beer and Pub Association, 2010).

If “accidental underestimation” of a self-defined usual glass is identified in the general population, this should be a priority for alcohol education initiatives and public health policy. This may also be 1 mechanism by which alcohol consumption is underreported in social surveys. This study is the first study exploring estimation accuracy in the general population, and the first in the United Kingdom.

## Materials and Methods

### Sites and Participants

A pilot study was conducted in May 2011 to test the questionnaire and study procedure. The results were used in the sample size calculation for the main study. Data were collected from 5 study sites in London and 1 in South East England on 12 occasions between July and October 2011 with a short break in August due to the London riots. Sites included shopping venues, drinking venues, and workplaces. Participants were a convenience sample of 283 adults (54% women) invited to take part in the study by a single researcher.

### Questionnaire

A brief self-completion questionnaire was administered to participants upon entering the study. The questionnaire contained demographic questions, and questions on quantity and frequency of alcohol consumption and alcohol unit awareness. The demographic questions and alcohol quantity and frequency questions were taken from the Health Survey for England (HSE) 2008 (NatCen Social Research, Royal Free and University College Medical School, 2008), and the alcohol unit awareness questions were taken from the HSE 2007 (the focus of which was “Healthy Lifestyles: Knowledge, Attitudes, and Behaviour”; NatCen Social Research, Royal Free and University College Medical School, 2007).

### Procedure

The majority of previous studies have used water as a substitute for alcohol (Carruthers and Binns, 1992; Kerr et al., 2005; Lemmens, 1994; White, 2005; White et al., 2003; Wilson, 1981), with 1 study using colored water (Wilkinson et al., 2011). Actual alcoholic beverages have been used in some studies (Banwell, 1999; Gill and Donaghy, 2004; Gill et al., 2007; Gual et al., 1999; Kerr et al., 2008). Real alcoholic drinks were used in this study rather than water or an imitation beverage as associated visual and olfactory cues may guide participants in pouring a glass most similar to that which they would pour at home. Beverages chosen were 2 wines (white wine at 12% ABV, red wine at 13.5% ABV), and 4 spirits (gin at 37.5% ABV, vodka at 37.5% ABV, whiskey at 40% ABV, and dark rum at 40% ABV). Participants were invited to choose both 1 wine and 1 spirit if they would usually drink these drinks at home.

The majority of previous studies either allowed respondents to use their own glasses (Banwell, 1999; Carruthers and Binns, 1992; Kerr et al., 2005; Lemmens, 1994; Wilkinson et al., 2011) or offered a selection as part of the research apparatus (Gual et al., 1999; White et al., 2003). One study asked respondents to point to a level on a marked vessel instead of pouring a glass themselves (Kaskutas and Graves, 2000). Two identical sets of 8 drinking glasses were used in this study. One set was used for light-colored drinks and the other set for dark-colored drinks so that residue in glasses did not affect the color of subsequent drinks poured. Once the questionnaire was completed, participants were asked to select a beverage. Participants were then asked to select a glass most similar to what they would use for that beverage at home. Participants were instructed to pour their “usual glass” and then to estimate the number of units poured (any whole number or decimal was accepted, fractions were recorded as decimals by the researcher). The poured drink was then measured using a funnel and graduated measuring cylinder and returned to the original bottle for re-use.

### Statistical Analysis

Wine and spirits are considered separately in the analyses of the pouring task as many (64%) participants poured both drinks. Outliers estimating their usual glass more than ±5 standard deviations from the mean were excluded. Correlation coefficients describe the relationship between estimated and actual units poured. Three categories of pouring accuracy were created using the difference between actual and perceived units poured: underestimators (greater than 0.5 units), correct estimators (±0.5 units), and overestimators (less than −0.5 units).

Multinomial (polytomous) logistic regression explored demographic and alcohol-related risk factors for underestimating or overestimating. Multinomial models presented are mutually adjusted. Likelihood ratio tests assessed the fit of the model with additional covariates. For wine pouring accuracy, the covariates investigated but which did not improve the fit of the model were income category, drinking frequency in the last year, number of drinking days in the last week, units consumed on heaviest drinking day in the last week, never drinking wine, drink drunk most often, and type of wine poured (red or white). For spirit pouring accuracy, the covariates investigated but not included were income category, employment status, drinking frequency in the last year, number of drinking days in the last week, never drinking spirits, drink type drunk most often, and type of spirit poured (gin, vodka, whiskey, or dark rum). The analyses were run on complete cases as there were a very small number (<5%) of missing values. All analyses were completed in Stata version 12 (StataCorp, College Station, TX).

## Results

### Descriptive Statistics

A total of 283 participants (54% women) completed the questionnaire and pouring task. The sample was relatively young, educated, and affluent compared with the general population (Table 1). Alcohol consumption was frequent and heavy with half of the sample drinking on 2 to 4 days in the last week and one-third reporting binge drinking (>8/6 U.K. units, 1 unit = 8 g ethanol [EtOH]) on their heaviest drinking day in the last week (Table 2).

**Table 1:** Sociodemographic Characteristics of Participants Completing Questionnaire and Pouring Task

	Men *n* (%)	Women *n* (%)	Total *n* (%)
Total sample	130 (100)	153 (100)	283 (100)
Age group			
16 to 34	73 (56.2)	83 (54.2)	156 (55.1)
35 to 54	42 (32.3)	52 (34.0)	94 (33.2)
55+	14 (10.8)	18 (11.8)	32 (11.3)
Ethnic group			
White	99 (76.2)	108 (70.6)	207 (73.1)
Any non-white	31 (23.8)	45 (29.4)	76 (26.9)
Highest educational qualification			
NVQ4/NVQ5/degree or equivalent	78 (60.0)	93 (60.8)	171 (60.4)
Higher education below degree	9 (6.9)	13 (8.5)	22 (7.8)
NVQ3/GCE A level equivalent	16 (12.3)	15 (9.8)	31 (11.0)
NVQ2/GCE O level equivalent	4 (3.1)	8 (5.2)	12 (4.2)
NVQ1/CSE other grade equivalent	6 (4.6)	7 (4.6)	13 (4.6)
Foreign/other	10 (7.7)	8 (5.2)	18 (6.4)
None	4 (3.1)	8 (5.2)	12 (4.2)
Employment status			
Employed	94 (72.3)	93 (60.8)	187 (66.1)
Unemployed	4 (3.1)	10 (6.5)	14 (4.9)
Unemployed and receiving benefits	0 (0.0)	8 (5.2)	8 (2.8)
Retired	8 (6.2)	4 (2.6)	12 (4.2)
Full-time education	23 (17.7)	38 (24.8)	61 (21.6)
Total household income^a^			
<£10,655.74	16 (12.3)	38 (24.8)	54 (19.1)
£10,655.75 to 16,900.00	5 (3.8)	18 (11.8)	23 (8.1)
£16,900.01 to 26,787.88	24 (18.5)	15 (9.8)	39 (13.8)
£26,787.89 to 41,864.41	27 (20.8)	29 (19.0)	56 (19.8)
>£41,864.42	52 (40.0)	51 (33.3)	103 (36.4)

a Based on income quintile thresholds in the HSE 2008.

There were a small number of missing values for each variable (<4%) which are not included in further analyses.

**Table 2:** Drinking Frequency and Consumption in the Last Week by Sex

	Men *n* (%)	Women *n* (%)	Total *n* (%)
Total sample	130 (100.0)	153 (100.0)	283 (100.0)
Ever drinks alcohol			
Yes	129 (99.2)	152 (99.3)	281 (99.3)
No	0 (0.0)	1 (0.7)	1 (0.4)
Ever pours alcoholic drinks for other people			
Yes	120 (92.3)	143 (93.5)	263 (92.9)
No	8 (6.2)	10 (6.5)	18 (6.4)
Drink drunk most often			
Any wine	38 (29.2)	82 (53.6)	120 (42.4)
Any beer/lager/cider shandy	41 (31.5)	15 (9.8)	56 (19.8)
Spirits or liqueurs	14 (10.8)	17 (11.1)	31 (11.0)
Other drinks	1 (0.8)	3 (2.0)	4 (1.4)
Two or more drink types	27 (20.8)	31 (20.3)	58 (20.5)
Drinking frequency (past 12 months)			
Almost every day	30 (23.1)	32 (20.9)	62 (21.9)
At least once a week	82 (63.1)	74 (48.4)	156 (55.1)
At least once a month	14 (10.8)	36 (23.5)	50 (17.7)
Less than once a month	4 (3.1)	11 (7.2)	15 (5.3)
Drinking days in the last week			
0 or 1	21 (16.2)	44 (28.8)	65 (23.0)
2 to 4	68 (52.3)	72 (47.1)	140 (49.5)
5 to 7	29 (22.3)	25 (16.3)	54 (19.1)
Units on heaviest drinking day in the last week			
Not applicable	2 (1.5)	6 (3.9)	8 (2.8)
Don't know	14 (10.8)	18 (11.8)	32 (11.3)
Less than recommended daily limits (<4/3 units)	22 (16.9)	45 (29.4)	67 (23.7)
Above daily limits, but below binge	33 (25.4)	33 (21.6)	66 (23.3)
Binge drinking (>8/6 units)	52 (40.0)	43 (28.1)	95 (33.6)

There were a small number of missing values for each variable (<5%) which were not included in further analyses.

The majority (95%, *n* = 270) of the sample reported they had heard of units, with half of those who reported not having heard of units subsequently estimating drinking guidelines or number of units in drinks. Awareness of drinking guidelines and number of units in certain drinks was similar to the equivalent sociodemographic group in HSE 2007 (data not shown, available on request from lead author).

Sixty-four percent of participants poured both wine and spirits. In total, 465 drinks were poured. The mean number of units poured of wine and spirits was 1.90 (SD 0.80, *n* = 264) and 1.93 (SD 0.78, *n* = 201), respectively. Mean number of units poured was similar for men and women and in each of the beverage subcategories.

The majority of participants poured more than 1 unit but less than the U.K. daily limits (4 units for men and 3 units for women, 1 U.K. unit = 8 g EtOH) as their usual glass for both wine (97% men and 78% women) and spirits (91% men and 78% women). Women were more likely than men to pour more than their daily limit as a usual glass for both wine (10% women vs. 0% men, *p* < 0.001) and spirits (7% women vs. 2% men, *p* = 0.097; *p*-values from chi-squared tests). One woman poured more than the binge drinking threshold (8 units for men and 6 units for women) as her usual glass.

Participants estimated the units of alcohol in their self-defined usual glass in 440 drinks (95% of total drinks poured). For wine, there was a moderate statistically significant (*r* = 0.48, *p* < 0.0001) correlation between estimated and actual units poured in a self-defined usual glass. This correlation was similar for spirits (*r* = 0.46, *p* < 0.0001). Although estimated and actual units poured of both wine and spirits are significantly correlated, the mean drink size differs across pouring accuracy groups for both beverages.

### Multivariate Analysis

Estimation in the 3 accuracy categories is shown in Table 3. For wine, 55% men and 50% women estimated their self-defined glass within half a unit. For spirits, 43% men and 40% women estimated within half a unit. Of the remainder, overestimation of a self-defined glass was more common than underestimation. A larger proportion of women than men underestimated poured units of both wines (23 and 14%, respectively) and spirits (21 and 12%). The mean difference between actual and perceived units poured was slightly but not significantly greater for overestimating than underestimating for both wine and spirits.

**Table 3:** Estimation Accuracy by Sex and Drink Type

Estimation accuracy	Wine			Spirits		
	
	Men	Women	Total	Men	Women	Total
Overall						
*n*	111	137	248	97	95	192
Mean diff	−0.34	−0.08	−0.20	−0.84	−0.43	−0.64
SD	1.03	1.00	1.02	1.61	1.31	1.48
Within 0.5 units						
*n*	61	68	129	42	38	80
%	55.0	49.6	52.0	43.3	40.0	41.7
Mean diff	0.02	0.00	0.01	0.00	0.00	0.00
SD	0.28	0.31	0.30	0.29	0.24	0.27
Underestimated by >0.5 units						
*n*	15	31	46	12	20	32
Percentage	13.5	22.6	18.5	12.4	21.1	16.7
Mean diff	0.77	1.11	1.00	0.80	1.02	0.94
SD	0.31	0.83	0.72	0.20	0.55	0.46
Overestimated by >0.5 units						
*n*	35	38	73	43	37	80
Percentage	31.5	27.7	29.4	44.3	38.9	41.7
Mean diff	−1.43	−1.20	−1.31	−2.12	−1.66	−1.91
SD	1.11	0.69	0.92	1.62	1.17	1.44

diff, difference.

The results of the multinomial logistic regression are shown in Table 4. Significant predictors of underestimation of a self-defined usual glass are the volume poured for both wine and spirits, and additionally for wine only belonging to a non-white ethnic group and being unemployed or retired. Not having a degree is a significant predictor of overestimation of a self-defined usual glass (of borderline significance for wine).

**Table 4:** Risk Factors for Underestimating and Overestimating Wines and Spirits from Multivariate Multinomial Logistic Regression

	Wine pouring accuracy (*n* = 248)		Spirit pouring accuracy(*n* = 192)	
		
	RRR	95% CI	RRR	95% CI
Within 0.5 units	1.00 (reference)		1.00 (reference)	
Underestimated by >0.5 units				
Sex (female)	1.68	0.76 to 3.69	2.03	0.72 to 5.71
Age 35 to 54 (vs. 16 to 34)	1.07	0.44 to 2.63	0.59	0.18 to 1.93
Age 55+ (vs. 16 to 34)	2.26	0.60 to 8.53	0.18	0.01 to 2.76
Volume poured (ml)	1.02	1.01 to 1.02**	1.04	1.01 to 1.06*
No degree (vs. degree or equivalent)	0.53	0.20 to 1.37	1.77	0.59 to 5.35
Non-white (vs. white)	3.88	1.65 to 9.16*	–	–
Unemployed or retired (vs. employed)	4.30	1.08 to 17.07*	–	–
Full-time student (vs. employed)	0.71	0.24 to 2.11	–	–
Drank >4/3 on heaviest day in last week	–	–	1.00	0.28 to 3.59
Drank >8/6 on heaviest day in last week	–	–	0.52	0.14 to 1.95
Overestimated by >0.5 units				
Sex (female)	1.08	0.58 to 1.99	0.92	0.44 to 1.92
Age 35 to 54 (vs. 16 to 34)	1.30	0.64 to 2.65	0.74	0.32 to 1.71
Age 55+ (vs. 16 to 34)	1.55	0.51 to 4.72	0.64	0.16 to 2.55
Volume poured (ml)	1.00	1.00 to 1.01	0.99	0.97 to 1.02
No degree (vs. degree or equivalent)	1.89	0.99 to 3.59*	2.78	1.28 to 6.07*
Non-white (vs. white)	1.44	0.68 to 3.02	–	–
Unemployed or retired (vs. employed)	2.40	0.70 to 8.20	–	–
Full-time student (vs. employed)	1.10	0.50 to 2.44	–	–
Drank >4/3 on heaviest day in last week	–	–	1.15	0.40 to 3.31
Drank >8/6 on heaviest day in last week	–	–	2.52	0.95 to 6.63

RRR, relative risk ratio; 95% CI, 95% confidence interval.

*p > 0.05, **p < 0.001.

Mutually adjusted. Slightly different models were constructed for wine and spirits based on the results of likelihood ratio tests. Covariates not included in the model are designated with dashes.

## Discussion

Although 95% of the sample had heard of units, estimation of the amount of alcohol in a self-defined usual glass was inaccurate with 58% spirit estimates, and 48% wine estimated greater than 0.5 units more or less than what was actually poured. More men and women overestimated than underestimated both wines and spirits (Table 3). Although these results are not generalizable and underestimation may be more frequent in a broader sample, our results suggest that accidental underestimation of the number of units in drinks poured at home is not able to contribute substantially to explaining the discrepancy between self-reported alcohol consumption and actual alcohol sales. This discrepancy amounts to around 40% of all the alcohol sold (average weekly alcohol consumption was 12.3 units per week per adult [16+] in the General Lifestyle Survey 2008 [Robinson and Bugler, 2008]. Alcohol sales were equivalent to 20.5 units per week per adult [16+] for the financial year 2008/2009 [HM Revenue and Customs, 2012]). Further research into alternative explanations (e.g., recall bias, nonresponse bias, sampling design) is required to understand this discrepancy. As inaccurate estimation is observed in this sample, it is hypothesized that in the general population estimation accuracy is likely to be lower than in this study. The demographic information collected shows the sample was relatively young, well educated, and affluent. Drinking was often frequent and/or heavy. Knowledge of drinking guidelines and units in standard drinks was more accurate in this group than the general population but similar for this sociodemographic group, based on nationally representative surveys (see HSE 2007 tables 7.9, 7.10, and 7.13–16; National Centre for Social Research and University College London, Department of Epidemiology and Public Health, 2007). Due to these factors, estimation of a self-defined usual glass is likely to be more accurate in this sample than the general population.

## Strengths

This is the first study in the United Kingdom which has measured both actual and perceived amounts of alcohol in a self-defined usual glass, and the first study in the general population (one previous study from Australia was conducted among 65- to 74-year-olds; Wilkinson et al., 2011). The questionnaire contained validated questions used in a nationally representative survey (HSE). The survey procedure was piloted. The sample size of this study is larger than several previous similar studies (Banwell, 1999; Gill and Donaghy, 2004; Gill et al., 2007; Kaskutas and Graves, 2000; White, 2005; White et al., 2003).

The detailed questionnaire design allowed for estimation accuracy to be explored with respect to sociodemographic and alcohol consumption variables in the multivariate analysis (see Materials and Methods). Likelihood ratio tests were used to examine the fit of the model with the inclusion of these variables. Variables that did not improve the fit of the model were not included in the final model. Only drinking above daily limits (>4/3 units) or binge drinking (>8/6 units) on the heaviest drinking day in the last week improved the fit of the model (for spirits only).

A particular strength of the study procedure is the use of actual alcoholic drinks so that visual and olfactory cues in pouring are not suppressed. A range of alcoholic drinks was provided so that participants could select a drink which they would usually pour. Original bottles were used so that participants could see the fraction of the bottle they had poured and look at the ABV on the label if they wished. A range of glasses was provided, which allowed participants to select what was most similar to what they would use at home and color contamination of drinks was avoided.

## Limitations

Due to the characteristics of the sample and the sample size (*n* = 283), the results of this study are not generalizable to the wider population. Researcher bias may have influenced the volume of a self-defined usual glass and the reported estimation of the amount of alcohol poured. There may have been deliberate overreporting where the reported estimated number of units may be greater than the actual estimated number of units due to conscious or unconscious concerns that the researcher may suggest the participant reduces their alcohol consumption. Similarly, participants who poured large glasses may have deliberately underestimated in a (conscious or unconscious) effort to define themselves as a lighter drinker. This bias was minimized by the researcher dressing casually and speaking to participants in an informal way but is difficult to fully overcome in a face-to-face survey. Future studies could consider triangulating estimation accuracy based on both prepoured and self-defined glasses to minimize any sense of a self-defined glass being reflective of the participant's alcohol consumption.

The participants in this study were or at least appeared sober. On the majority of occasions that individuals pour alcoholic drinks, they will also be consuming alcohol. It is probable that the volume of a self-defined “usual glass” and the perception of the amount of alcohol in that glass will change with increasing levels of intoxication. One hypothesis is that as an individual becomes intoxicated that the volume poured increases and that the propensity to underestimate increases. This would contribute to explaining the discrepancy between self-reported alcohol consumption and actual alcohol sales.

A procedural limitation is that ice cubes were not available to participants pouring spirits. Some previous studies conducted in participants' own homes have allowed for ice when considering spirits, by either allowing for ice melt in volume calculations (Kerr et al., 2005) or using plastic ice rocks (Wilkinson et al., 2011). Use of plastic ice rocks was considered, but it was decided that these are not a perfect substitute for real ice due to their often large size. Instead participants were asked to imagine that they were going to add ice afterward.

## Conclusion

Future studies investigating a self-defined usual glass of alcoholic beverages should consider recruiting intoxicated individuals, although other challenges should be expected. The validity of questionnaire responses may decrease and there would be ethical considerations recruiting intoxicated individuals. Additionally, as this study was not able to provide evidence for the systematic underestimation of the amount of alcohol in a self-defined usual glass, different mechanisms by which alcohol consumption is underreported in social surveys should be investigated further using qualitative and quantitative methods.

As participants underestimated pour larger measures on average than those who were correct or those who overestimated, a possible policy recommendation is to advocate pouring smaller measures to reduce the risk of underestimation. Previous calls have been made for a half-size bottle of wine (375 ml) to be more widely available in the off-trade to reduce alcohol consumption (Groves, 2008). This study supports this call as this may also promote a reduction in the size of a “usual glass,” leading to a reduction in the likelihood of underestimating the poured drink.
